# Evaluation of the RESIST ACINETO multiplex immunochromatographic assay for detection of OXA-23-like, OXA-40/58-like and NDM carbapenemase production in *Acinetobacter baumannii*

**DOI:** 10.1093/jac/dkad253

**Published:** 2023-08-11

**Authors:** Stefano Mancini, Helena M B Seth-Smith, Natalia Kolesnik-Goldmann, Vladimira Hinic, Tim Roloff, Frank Imkamp, Adrian Egli

**Affiliations:** Institute of Medical Microbiology, University Zurich, Gloriastrasse 28/30, 8006 Zurich, Switzerland; Institute of Medical Microbiology, University Zurich, Gloriastrasse 28/30, 8006 Zurich, Switzerland; Institute of Medical Microbiology, University Zurich, Gloriastrasse 28/30, 8006 Zurich, Switzerland; Institute of Medical Microbiology, University Zurich, Gloriastrasse 28/30, 8006 Zurich, Switzerland; Institute of Medical Microbiology, University Zurich, Gloriastrasse 28/30, 8006 Zurich, Switzerland; Institute of Medical Microbiology, University Zurich, Gloriastrasse 28/30, 8006 Zurich, Switzerland; Institute of Medical Microbiology, University Zurich, Gloriastrasse 28/30, 8006 Zurich, Switzerland

*Acinetobacter baumannii* is a major cause of hospital-acquired infections and among the top five pathogens associated with mortality.^1^ Due to its ability to rapidly acquire antimicrobial resistance traits, MDR isolates have been reported worldwide. Carbapenem-resistant *A. baumannii* (CRAB) are a particular concern, as only few treatment options, including colistin, tigecycline/eravacycline and cefiderocol, are currently available. For this reason, this pathogen has been listed by the WHO as ‘priority 1’ pathogen for research of new antimicrobials.^2^ In this context, rapid diagnostics is crucial to guide best antibiotic treatment^3^ and to prevent nosocomial transmission of CRAB. The most prevalent acquired carbapenemases in *A. baumannii* are class D oxacillinases, including the OXA-23, OXA-40 and OXA-58 groups. Other less frequently acquired carbapenemases include class A (e.g. carbapenemase variants of GES-type) and class B MBLs (e.g. NDM, VIM and IMP). Existing phenotypic methods are quite labour-intensive and exhibit variable performances in detecting carbapenemase production in *A. baumannii*.^4,5^ Molecular methods including PCR or loop-mediated isothermal amplification (LAMP) assays allow for accurate detection of most prevalent carbapenemase genes but require expensive equipment.^6^ Isothermal detection methods combined with lateral flow strips have been recently developed for rapid detection of the most prevalent carbapenemase genes in *A. baumannii*, but currently these assays are not commercially available.^7^ Immunochromatographic lateral flow assays (LFIAs) for the detection of carbapenemase-producing *A. baumannii* isolates are available on the market, but only allow detection of single carbapenemase types, such as OXA-23 (OXA-23 *K*-SeT, Coris BioConcept, Belgium) or metallo-carbapenemases (NG-Biotech, France).^8^ A recently developed type of LFIA for rapid detection of the most prevalent acquired carbapenemases in *A. baumannii*, including OXA-23, OXA-40/58 and NDM-types, is the ‘RESIST ACINETO’ assay (Coris BioConcept, Belgium).^9^ It is important here to note that although OXA-40 and OXA-58 belong to different families of OXA carbapenemases, their detection is combined in a single band and thus cannot be distinguished. This may represent a drawback for tracking certain types of outbreaks.

**Figure 1.**
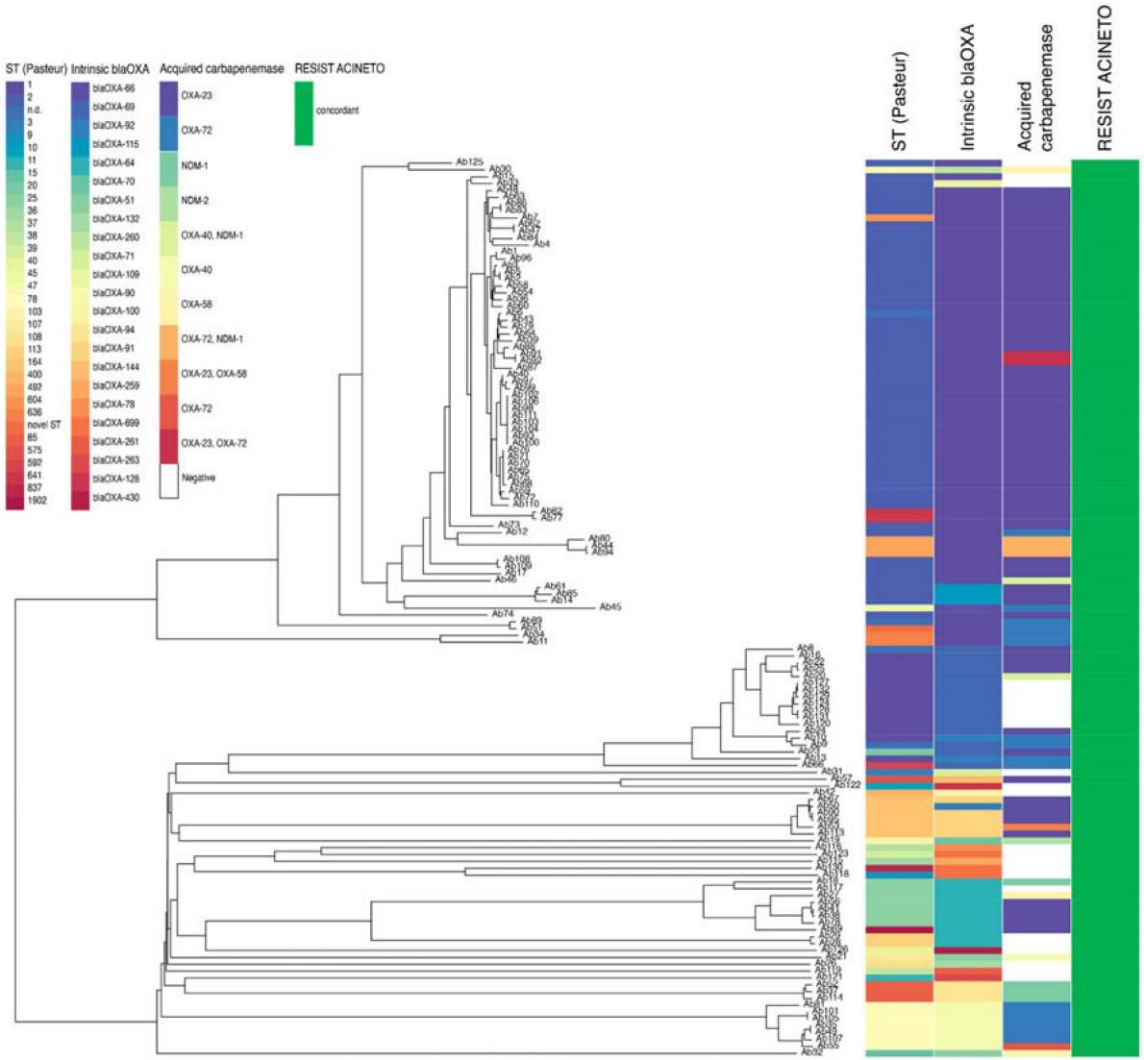
Phylogenetic neighbour-joining tree of *A. baumannii*. The tree was generated in Ridom SeqSphere+ based on core genes with associated metadata in columns, from left to right: ST (Pasteur), intrinsic oxacillinases identified in the genome, acquired carbapenemases and RESIST ACINETO result (green for congruent result).

Here we evaluate retrospectively the diagnostic performance of this new assay using a collection of 131 *A. baumannii* clinical isolates (Figure 1). Fourteen strains were obtained from the Institut Pasteur’s strain collection (https://www.pasteur.fr/en/public-health/biobanks-and-collections/collection-institut-pasteur-cip), while the remaining 117 clinical isolates were derived from single patients between January 2014 and December 2022 in the routine diagnostic laboratory of the Institute of Medical Microbiology at the University of Zurich. Of these, 106 exhibited carbapenem-resistant profiles, while 25 were susceptible to carbapenems. β-Lactamase-genes were detected by WGS, which was performed using our in-house available Illumina MiSeq platform with paired-end 150-nt reads. Intrinsic oxacillinases, as well as acquired carbapenem resistance markers, including carbapenemases and ESBLs, were detected using Unicycler v0.4.8 assemblies^10^ in combination with ABRicate (https://github.com/tseemann/abricate) and the NCBI database.^11^ All strains were typed in Ridom SeqSphere+ by MLST according to the Pasteur (ST) scheme and in addition with core-genome MLST.^12^ RESIST ACINETO was performed on isolated colonies grown overnight on blood agar plates (tryptic soy agar with 5% sheep blood, bioMérieux, France) at 37°C according to the manufacturer’s instructions. All genomes were submitted to the ENA (https://www.ebi.ac.uk/ena/browser) under project number PRJEB62871.

The strain collection comprised 25 carbapenemase-negative and 106 carbapenemase-producing isolates. Seventy-two of 106 isolates harboured *bla*_OXA-23_ (68%), 17 *bla*_OXA-72_ (16%), three *bla*_OXA-58_ and one *bla*_OXA-40_, while three isolates carried two oxacillinase genes (two *bla*_OXA-23_/*bla*_OXA-58_ and one *bla*_OXA-23_/*bla*_OXA-72_). Four isolates harboured *bla*_NDM-1_ and one *bla*_NDM-2_, while the remaining carbapenemase producers harboured a combination of genes coding for NDM-1 and an oxacillinase (two *bla*_NDM-1_/*bla*_OXA-23_, three *bla*_NDM-1_/*bla*_OXA-72)_. The *A. baumannii* isolates belonged to 34 different STs, with ST2 being the most prevalent (58/131; 36.7%).

RESIST ACINETO correctly identified all six carbapenemase variants, including those from the isolates producing two carbapenemases, thus exhibiting excellent sensitivity (100%). Strong bands appeared within 5–10 min of incubation in all but one case, where a faint band corresponding to NDM emerged at 15 min incubation. Nonetheless this isolate was classified as a true positive. OXA-72 was identified as a member of the OXA-40 group of OXA β-lactamases. No false positive results, which might also arise due to cross-reactivity with one of the 23 detected intrinsic OXA-51-like oxacillinases, among which OXA-66 was the most prevalent (66/131, 50.4%), were observed (specificity 100%).

A limitation of our study is that the collection of *A. baumannii* isolates is biased and reflects the epidemiological situation of the Zurich region in Switzerland, with only six different carbapenemase variants identified so far. Also, while some types were abundantly present, such as OXA-23 (68%), other globally present types, such as OXA-40, were underrepresented (1%). Moreover, in this study the performance of the RESIST ACINETO was tested on *A. baumannii* colonies grown on blood agar plates. Considering that most laboratories identify *A. baumannii* on Columbia agar or MacConkey agar plates, further studies with a more diverse collection of carbapenemase variants and isolates grown on different media are warranted to fully evaluate the robustness of the method.

In conclusion, RESIST ACINETO provides a reliable test for the detection of the most prevalent carbapenemases in *A. baumannii*. The sensitivity and specificity from isolated colonies of overnight growth is excellent (each 100%).
